# Serotypes and Antimicrobial Resistance in *Salmonella enterica* Recovered from Clinical Samples from Cattle and Swine in Minnesota, 2006 to 2015

**DOI:** 10.1371/journal.pone.0168016

**Published:** 2016-12-09

**Authors:** Samuel Hong, Albert Rovira, Peter Davies, Christina Ahlstrom, Petra Muellner, Aaron Rendahl, Karen Olsen, Jeff B. Bender, Scott Wells, Andres Perez, Julio Alvarez

**Affiliations:** 1 Department of Veterinary Population Medicine, College of Veterinary Medicine, University of Minnesota, St Paul, MN, United States of America; 2 Veterinary Diagnostic Laboratory, College of Veterinary Medicine, University of Minnesota, St Paul, MN, United States of America; 3 Epi-interactive, Wellington, New Zealand; 4 School of Statistics, University of Minnesota, Minneapolis, United States of America; Wadsworth Center, UNITED STATES

## Abstract

Salmonellosis remains one of the leading causes of foodborne disease worldwide despite preventive efforts at various stages of the food production chain. The emergence of multi-drug resistant (MDR) non-typhoidal *Salmonella enterica* represents an additional challenge for public health authorities. Food animals are considered a major reservoir and potential source of foodborne salmonellosis; thus, monitoring of *Salmonella* strains in livestock may help to detect emergence of new serotypes/MDR phenotypes and to gain a better understanding of *Salmonella* epidemiology. For this reason, we analyzed trends over a nine-year period in serotypes, and antimicrobial resistance, of *Salmonella* isolates recovered at the Minnesota Veterinary Diagnostic Laboratory (MVDL) from swine (n = 2,537) and cattle (n = 1,028) samples. Prevalence of predominant serotypes changed over time; in swine, *S*. Typhimurium and *S*. Derby decreased and *S*. Agona and *S*. 4,5,12:i:- increased throughout the study period. In cattle, *S*. Dublin, *S*. Montevideo and *S*. Cerro increased and *S*. Muenster became less frequent. Median minimum inhibitory concentration (MIC) values and proportion of antibiotic resistant isolates were higher for those recovered from swine compared with cattle, and were particularly high for certain antibiotic-serotype combinations. The proportion of resistant swine isolates was also higher than observed in the NARMS data, probably due to the different cohort of animals represented in each dataset. Results provide insight into the dynamics of antimicrobial resistant *Salmonella* in livestock in Minnesota, and can help to monitor emerging trends in antimicrobial resistance.

## Introduction

Non-typhoidal *Salmonella enterica* is a leading cause of foodborne disease in both developing and developed countries [1]. Prevention of salmonellosis (caused by nontyphoidal *Salmonellae*) is challenging due to its complex epidemiology and multiple modes of transmission. Most food (poultry, swine, cattle, sheep) and companion (reptiles, dogs) animal species are potential reservoirs and sources of infection, and *S*. *enterica* may be isolated from both healthy and clinically affected animals. Despite substantial efforts to prevent foodborne salmonellosis in developed countries, incidence of human illness has remained constant, although the relative importance of serotypes fluctuates over time [2]. Emergence of multiple antibiotic resistant *Salmonella* adds another important dimension to the challenge of *Salmonella* control as resistant variants can compromise the ability to treat human infections, an issue of particular importance in the case of systemic infections [3]. Since the 1990s, resistance to many conventional antibiotics, such as ampicillin, chloramphenicol, and trimethoprim/sulfonamides, became relatively common in clinical isolates, thus posing an additional challenge due to the emergence of multiple resistant phenotypes [4]. Though in the last 20 years the proportion of *Salmonella* isolates resistant to at least one antibiotic group recovered from clinical human samples has decreased [5, 6], drug-resistant non-typhoidal *Salmonella* is still considered a serious threat in the U.S. [7].

Use of antimicrobials in both human medicine and food production is considered a major factor increasing emergence of antimicrobial resistant bacteria, but the role that prophylactic, therapeutic or subtherapeutic use of antibiotics in food animals has in the current situation is yet-to-be elucidated [8]. Nevertheless, veterinary use of antimicrobials is considered a key factor in the emergence of antimicrobial-resistant *Salmonella*, and multiple resistance to antimicrobials is more commonly observed in isolates from food animals than from human clinical cases [3, 9, 10]. In order to monitor the trends in the occurrence of antimicrobial resistance (AMR) in food animals, a number of countries have launched national surveillance programs [9]. In the U.S. the National Antimicrobial Resistance Monitoring System (NARMS) combines information on AMR in *Salmonella* isolates recovered from different sources, including food animals sampled at the slaughterhouse and, until 2005–2006, isolates recovered from clinically affected animals submitted by certain State veterinary laboratories and National Veterinary Services Laboratory (NVSL) [11]. Alternatively, information on most common serotypes in food animals in the U.S. can be also obtained from the USDA NVSL in Ames, IA, which performs serotyping on most of the veterinary isolates recovered from clinical (clinically affected animals) and non-clinical samples (from herd monitoring and surveillance activities) submitted by veterinary diagnostic laboratories. Analysis of the NARMS and NVSL information on *Salmonella* isolates recovered from food animals reveals that even though certain serotypes are usually associated with certain animal species, shifts in predominant serotypes and changes in the frequency of presentation of antimicrobial resistance to several antibiotics occur over time, thus highlighting the usefulness of these data for surveillance and monitoring purposes [12–15]

Here, we described the evolution in the frequency of different *Salmonella* serotypes among isolates recovered from samples collected from cattle and swine in Minnesota submitted to the Minnesota Veterinary Diagnostic Laboratory (MVDL) over a nine year period to i) assess the occurrence of significant changes in their distribution; ii) detect trends in the presentation of resistance phenotypes; and iii) compare results obtained from swine and cattle located in Minnesota to those reported at the national level through the NARMS data. Results can help inform management and policy decisions on surveillance and control of *Salmonella* in food animals, and may provide information on emergent serotypes/phenotypes that could be useful from a public health perspective.

## Material and Methods

### Bacterial collection and laboratory analysis

All *Salmonella* spp. isolates recovered from cattle and swine samples from July 2006 through June 2015 at the MVDL were included in the study. The MVDL, located at the University of Minnesota in St Paul, Minnesota, receives animal diagnostic submissions predominantly from farms within the state of Minnesota and ranks #3 in swine production and #6 in dairy production among all states in the country. Between 2006 and 2014, the MVDL received between 14,000 and 25,000 cattle and swine samples per year from within Minnesota for culture of intestinal bacteria. All animal samples included in this study were originally sent to the Veterinary Diagnostic Laboratory for diagnostic purposes, and thus no experimental research on animals was performed.

Samples submitted for isolation of *Salmonella* are routinely processed following protocols described elsewhere [16]: clinical samples are inoculated onto solid media (sheep blood agar, MacConkey agar and brilliant green agar) and incubated for 18–24 hours at 37°C, and onto a selective enrichment medium (Hajna’s tetrathionate broth) that is incubated for 18–24 hours at 42°C. The broth is then plated onto brilliant green agar and xylose lysine deoxycholate agar and incubated for 18–24 hours at 37°C. Occasionally only brilliant green agar is used for the direct plating isolation. During the period 2011–2014, the routine use of enrichment broth for culture was discontinued so that it was not used in all samples.

When colonies with morphology consistent with *Salmonella* were observed, one CFU belonging to each morphology identified in each processed sample was analyzed to determine its serogroup using *Salmonella* O (Becton Dickinson, Franklin Lanes, New Jersey, US), and one isolate of each serogroup detected in an accession (that may include one or more samples from one or more animal submitted in the same shipment) was then submitted for serotyping at the National Veterinary Services Laboratory (NVSL) in Ames, IA. Antibiotic sensitivity testing was performed at the MVDL using the Sensititre automatized dilution system (Trek Diagnostic Systems, Cleveland, OH) to determine the minimum inhibitory concentration (MIC). Only antibiotics that were tested in >40 isolates every year were considered in this analysis. AMR against ampicillin (A), ceftiofur (C), florfenicol (F), neomycin (N), oxytetracycline (O), spectinomycin (Sp), sulfadimethoxine (Sul), and trimethoprim-sulfamethoxazole (Ts) was tested in all isolates. Additionally, for the swine isolates susceptibility against gentamicin (G) and enrofloxacin (E) was also tested in all and 87% of the total number recovered, respectively. Minimum inhibitory concentrations (MICs) for each antimicrobial were analyzed as a semi-quantitative variable and also translated into a qualitative scale (susceptible, intermediate, resistant) using previously internal (N, Sul) or, when available, CLSI established break-points for *Salmonella* derived from animals (C, E, F) and humans (A, G, O, Ts) [17] (Table 1).

**Table 1:** Criteria used for qualitative assessment of the susceptibility of *Salmonella* isolates recovered from cattle and swine samples for antimicrobials in which resistant isolates were found.

Antimicrobial	MIC Cut-offs Values			Reference
	Susceptible	Intermediate	Resistant	
Ampicillin (A)	≤8	16	≥32	[17]
Ceftiofur (C)	≤2	4	≥8	[17]
Enrofloxacin (E)	≤0.25	0.5	≥1	[17]
Florfenicol (F)	≤4	8	≥8	[17]
Gentamicin (G)	≤4	8	≥16	[17]
Neomycin (N)	≤8		≥16	Internal
Oxytetracycline (O)	≤4	8	≥16	[17]
Spectinomycin (S)	NA^a^	NA^a^	NA^a^	NA^a^
Sulfadimethoxine (Sul)	≤256		≥512	Internal
Trimethoprim/ Sulfamethoxazole (Ts)	≤2		≥4	[17]

^a^NA: not available

### Data analysis

In order to avoid overrepresentation of isolates recovered from farms submitting frequently within a short period (here a month), the epidemiological unit for analysis was the isolate (defined as an individual serotype and resistotype) recovered per farm and month-year. The farm/month-year was a spatio-temporal unit determined by three attributes, namely, the (i) submitting client (ii) location, and (iii) date (month and year). Information on those three fields for each isolate was recovered from the MVDL database and stored in standard software for database management, to remove isolates with the same serotype and AMR pattern recovered from the same farm in the same month from the same fiscal year (defined as a 12 month period starting from July 1 to June 30 of the following calendar year). Data wrangling process was performed in Python using the xlrd ver 0.9.3 and simplejson ver 3.8.1 packages in order to create a local NoSQL database (MongoDB). Data aggregation was performed in Python using the pymongo MongoDB connector.

The number of farms submitting samples from which at least one *Salmonella* isolate was recovered was computed by year and animal species. Proportions of isolates (isolates per farm/month-year, as defined above) belonging to different serotypes as well as proportion of isolates resistant to each antibiotic were then determined. Changes in the prevalence of serotypes for each host species over time were evaluated using a chi-square test for trends in which the proportions of isolates belonging to a given serotype recovered in each year during the study period were alternatively evaluated to evaluate if there was an increasing, decreasing or no trend.

For the antimicrobial data, first distributions of MIC values obtained in the different years for each antimicrobial were compared visually and more formally using a non-parametric tests (Kruskal-Wallis test) in which the MIC was the semi-quantitative variable being compared between years (independent variable). Finally, the evolution of MICs over time in each species was evaluated using a cumulative logit model, so that

$$logit[P(Y\leq z_{j}|t_{i}]=\alpha_{0j}+\alpha_{1}t_{i}$$

With *z*_*j*_ representing the MIC category *j* and *t*_*i*_ a year in the study period, so that each cumulative logit has its own intercept *α*_0*j*_ increasing in j (MIC categories from low to high) and all share the same time effect *α*_1_, that estimates the temporal trend during the study period [18]. Proportional odds assumption was assessed by comparing the log likelihood of the ordered logit model with a multinomial logit model, in which a different *α*_1*j*_ time-specific coefficient was estimated for each cumulative logit, using a likelihood ratio test [19]. When a p-value<0.05 was found, the time-associated coefficient *α*_1*j*_ was estimated separately for MICs in which a positive or negative value (suggesting an increasing or decreasing trend for those particular MICs) had been obtained in the multinomial model. These semiquantitative analyses of MICs obtained against Sul and Ts were not performed because only both antimicrobials were tested at only two concentrations.

Subsequently, MICs were transformed into a binary variable (resistant/non-resistant with susceptible including both susceptible and intermediate isolates) for all antimicrobials except Sp. A chi-square test was then performed to test for trends in the prevalence of resistant phenotypes among cattle and swine isolates over time. For those antimicrobials in which significant results were obtained, the yearly rate of change in the proportion of resistant isolates along with its 95% confidence interval was calculated using a linear regression model in which the log-transformed proportion of resistant isolates (π) and time (*t*_*i*_) were the dependent and independent variables, respectively, so that

$$log(\pi_{i})=\alpha +\beta t_{i}$$

and the relative change per year was computed as 100 * (*e*^*β*^ − 1). The Cochrane–Orcutt procedure was applied to control serial correlation [20, 21].

Additionally, the 9-year cumulative percentage of isolates resistant to each antibiotic was calculated for each host species. A pairwise chi-square test was used to compare the proportion of resistant isolates across serotypes for the most prevalent (accounting for >50% of all isolates recovered through the study period) serotypes. The statistical tests were performed using the stats package in R [22] and the WINPEPI 9.4 software, and a p-value below 0.05 was considered significant in all tests [23].

Finally, rarefaction analysis was performed to estimate the resistotype richness in bovine and porcine isolates, controlling for the different number of samples obtained from each source. Resistotypes were defined by susceptibility (susceptible, intermediate and resistant) to the seven antimicrobials that were tested in all isolates of both swine and cattle origin. Simpson’s index of diversity was calculated for each source to capture the diversity of resistotypes in both sources, and to extend the analysis beyond separate assessments of resistotype richness (i.e. the number of resistotypes detected in each source) to ultimately also capture relative resistotype abundance, i.e., how common or rare a resistotype is relative to others. The function RAREFY in the package VEGAN (version 2.2–3) was implemented in R (version 3.2.3) for rarefaction calculations and diversity was calculated in Past (version 3.10) [24]. Values with non-overlapping 95% confidence intervals were considered statistically significant.

To capture the similarity of resistotypes detected in bovine and porcine sources, the proportional similarity index (PSI), or Czekanowski index, was used to measure the area of overlap of the two frequency distributions of isolates resistant to each of the seven core antimicrobials per source.

Furthermore, the PSI was used to investigate the similarity of resistotypes between the most common serotypes of both porcine and bovine origin. Bootstrap confidence intervals were estimated as described previously [25]. The PSI is an objective and simple estimate of the area of intersection between two frequency distributions [26]. The PSI estimates the similarity between the frequency distributions of i.e. bacterial sub types between different reservoirs. It is calculated by:

$$PSI=1-0.5\sum_{i}\left|p_{i}-q_{i}\right|=\sum_{i}min(p_{i},q_{i})$$

where pi and qi represent the proportion of strains belonging to type i out of all strains typed from species P and Q [26, 27]. The values for PSI range from 1 for the highest possible similarity to 0 for distribution with no common types. Bootstrap confidence intervals for this measure can be estimated based on the approach applied by Garrett et al [28]. This technique has also recently applied to support source attribution studies of human campylobacteriosis [25, 29].

## Results

### Swine

**Fig 1:**
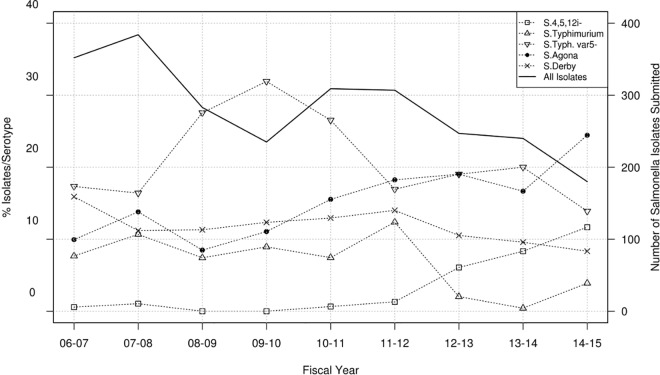
*Salmonella* serotypes recovered from swine samples at the MVDL in 2006–2015. Number of isolates with a given serotype recovered from swine samples per year (solid line, right axis) and percentage of these isolates belonging to the four predominant serotypes plus the *Salmonella* I 4,5,12:i:- serotype identified per year (dotted lines, left axis).

The mean number of *Salmonella* isolates recovered from swine submissions to the MVDL every fiscal year was 281 (range 180–384), and decreased non-monotonically over the study period (Fig 1). A total of 2,644 *Salmonella* isolates were cultured from samples from 1,832 swine farms in Minnesota between 2006 and 2014. A total of 2,464 farm/month-years were identified over that period as defined above. Of those, 60 farm/month-years (2.4%) that yielded 104 isolates were excluded because susceptibility testing had not been performed. After exclusion of multiple isolates submitted from the same farm within the same fiscal month-year, 2,537 *Salmonella* isolates were included in the analysis. Of these, 87% (2215/2537) were tested against enrofloxacin. Isolates were recovered primarily from tissue (85% from intestine, liver, lung, lymph nodes and occasionally other tissues) and fecal samples/swabs (14%).

A total of 79 different serotypes were identified among the 2,513 isolates that were successfully serotyped (in 24 cases, multiple serotypes were found or the isolate was considered untypable) (S1 Table). The four most prevalent serotypes recovered from swine at the farm/month-year level accounted for >50% of all isolates and included: *S*. Typhimurium var 5- (n = 531, 20.9%), *S*. Agona (n = 373, 14.7%), *S*. Derby (n = 307, 12.1%), and *S*. Typhimurium (n = 184, 7.3%). The frequency of detection of these organisms varied across years (Fig 1). A significant (p<0.001) increase in serotypes 4,5,12:i:- and Agona was detected, while *S*. Typhimurium and *S*. Derby decreased (p<0.05). At the farm level, there was a notable increase in the occurrence of serotype 4,5,12:i:- from < 1% of *Salmonella* positive farms in years 2011–2012 to 13.9% of farms in 2014–2015 (Fig 1).

Significant (Kruskal-Wallis test, p<0.05) differences in the MIC values recorded each year were found in all antimicrobials evaluated except G and N, though statistically significant differences between years were only found in post-hoc tests for A, E, O and Sp. Changes over time in the proportion of swine *Salmonella* isolates belonging to each MIC are depicted in S1 Fig. In the MIC cumulative logit models, a moderate but significant (p<0.01) decreasing time trend was observed for A, C, F, O and Sp (for MICs above 16 μg/ml), while a highly significant (p<0.001) increasing trend was obtained for the E data (Table 2).

**Table 2:** Time associated odds ratio quantifying the change in the probability of obtaining a response equal to or lower than each MIC level measured for each antimicrobial per year obtained using a cumulative logistic regression model (values below 1 indicate a decreasing probability of finding isolates with lower MIC values).

Antimicrobial	Swine			Cattle		
	Model	OR (p-value)	95% CI	Model	OR (p-value)	95% CI
Ampicillin	*P*(*Y* ≤ *MIC*_*j*_|*year*_*i*_)	**1.007 (<0.001)**	**1.042–1.114**	*P*(*Y* ≤ *MIC*_*j*_|*year*_*i*_)	0.921 (0.13)	0.921–1.010
Ceftiofur	*P*(*Y* ≤ *MIC*_*j*_|*year*_*i*_)	**1.050 (0.001)**	**1.020–1.081**	*P*(*Y* ≤ *MIC*_1_|*year*_*i*_)^a^	**1.126 (<0.001)**	1.061–1.195
				*P*(*Y* ≤ *MIC*_2:*j*_|*year*_*i*_)^a^	**0.927 (0.002)**	0.883–0.973
Enrofloxacin	*P*(*Y* ≤ *MIC*_*j*_|*year*_*i*_)	**0.721 (<0.001)**	**0.681–0.764**	NA	NA	NA
Florfenicol	*P*(*Y* ≤ *MIC*_*j*_|*year*_*i*_)	**1.068 (<0.001)**	**1.037–1.099**	*P*(*Y* ≤ *MIC*_1:2_|*year*_*i*_) ^b^	1.045 (0.11)	0.991–1.103
				*P*(*Y* ≤ *MIC*_3:*j*_|*year*_*i*_) ^b^	**0.951 (0.040)**	0.905–0.998
Gentamicin	*P*(*Y* ≤ *MIC*_*j*_|*year*_*i*_)	1.009 (0.56)	0.978–1.041	NA	NA	NA
Neomycin	*P*(*Y* ≤ *MIC*_*j*_|*year*_*i*_)	1.020 (0.21)	0.989–1.052	*P*(*Y* ≤ *MIC*_*j*_|*year*_*i*_)	**1.089 (0.002)**	**1.030–1.151**
Oxytetracycline	*P*(*Y* ≤ *MIC*_1_|*year*_*i*_) ^c^	0.719 (0.18)	0.444–1.162	*P*(*Y* ≤ *MIC*_1_|*year*_*i*_) ^d^	**1.691 (<0.001)**	**1.242–2.301**
	*P*(*Y* ≤ *MIC*_2:*j*_|*year*_*i*_) ^c^	**1.113 (<0.001)**	**1.060–1.169**	*P*(*Y* ≤ *MIC*_2:*j*_|*year*_*i*_)^d^	0.968 (0.17)	0.924–1.014
Spectinomycin	*P*(*Y* ≤ *MIC*_1_|*year*_*i*_) ^e^	0.954 (0.66)	0.775–1.175	*P*(*Y* ≤ *MIC*_*j*_|*year*_*i*_)	**1.236 (<0.001)**	**1.178–1.296**
	*P*(*Y* ≤ *MIC*_2:*j*_|*year*_*i*_)	**1.056 (<0.001)**	**1.024–1.089**			

^a^ MIC_1_ = ≤0.5 μg/ml; MIC_2:j_ = 1, 2, 4 and 8 μg/ml.

^b^ MIC_1:2_ = ≤1 and 2 μg/ml; MIC_3:j_ = 4 and 8 μg/ml.

^c^ MIC_1_ = ≤0.5 μg/ml; MIC_2:j_ = 1, 2 and 4 μg/ml.

^d^ MIC_1_ = ≤0.5 μg/ml; MIC_2:j_ = 1, 2 and 4 μg/ml.

^e^ MIC_1_ = ≤16 μg/ml; MIC_2:j_ = 32 and 64 μg/ml.

**Fig 2:**
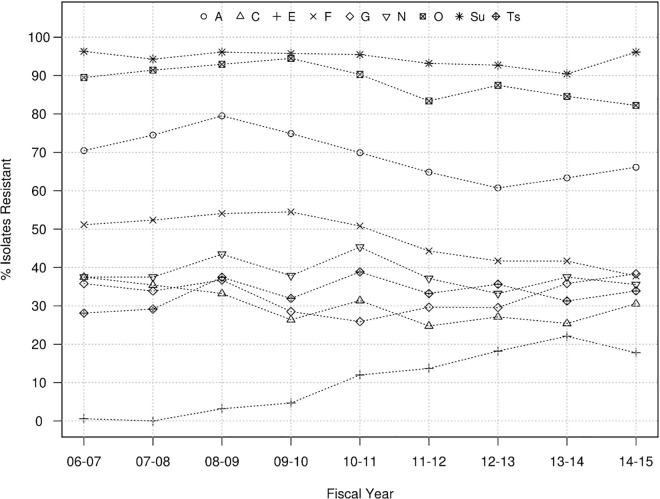
Evolution in antimicrobial resistant *Salmonella* isolates recovered from swine at the MVDL in 2006–2015. Proportion of *Salmonella* isolates recovered from swine samples that were resistant to ampicillin (A), ceftiofur (C), enrofloxacin (E), florfenicol (F), gentamicin (G), neomycin (N), oxytetracycline (O), sulfadimethoxine (Sul), spectomycin (Sp) and trimethorpim/ sulfamethoxazole (Ts).

**Fig 3:**
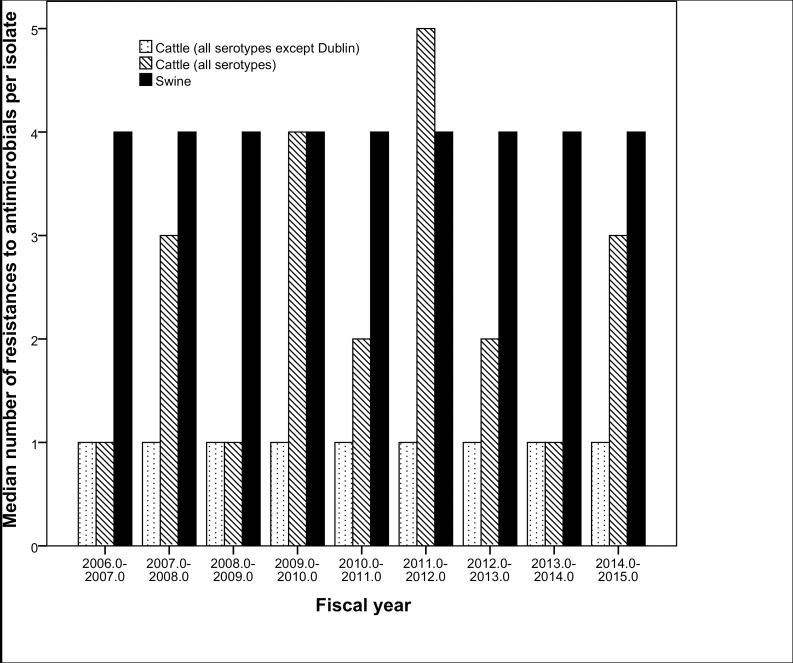
Median number of antimicrobials to which isolates recovered from swine (black bars) and cattle (dashed bars) samples were resistant to per year.

From a qualitative standpoint, the proportions of isolates resistant to other antibiotics over the years ranged between 60%-97% for A, O and Sul, and 25–55% for C, F, G, N and Ts, while proportions below 25% were observed for E (Fig 2). Overall no significant temporal trends were identified in the proportion of swine *Salmonella* isolates resistant to G, N and Ts while there were significant (p<0.05) though moderate (<6.5% annual rate of decrease) decreasing trends for resistance to A, C, F, O, and Sul (Table 3 and Fig 2). The only antibiotic for which a significant (P<0.001) increasing trend in the proportion of resistant isolates was observed was E, with an increase from 0% to 18% between 2006 and 2014 (Table 3 and Fig 2). Overall, tested isolates were resistant to a median number of four antimicrobials and 4.3% (n = 108) of them were resistant to all nine antimicrobials for which cut-off values were available. If E and G are not considered (so that the same panel of antimicrobials used in cattle is considered), the overall mean and annual median number of resistances per isolate was still 4 throughout the whole period (Fig 3). The most prevalent resistotype (R1) was O-Sul, which was found in 11.7% (298/2,537) of all swine isolates followed by resistotypes (R2) A-F-O-Sul (n = 279, 11%) and resistotype (R3) A-C-F-G-N-O-Sul-Ts (n = 213, 8.4%). Resistance to R2 was particularly prevalent among *S*. Typhimurium (78/184) and *S*. Typhimurium var. 5- (192/531) serotypes.

**Table 3:** Number and proportion of *Salmonella* isolates resistant to different antimicrobials recovered from swine and cattle samples between 2006 and 2015.

Antimicrobial^a^	Swine (n = 2,537)			Cattle (n = 1,167)		
	Resistant strains	% resistant	06–15 trend (95% CI)^b^	Resistant strains	% resistant	06–15 trend (95% CI)^b^
Ampicillin (A)	1771	69.8%	**-3.26 (-5.5 to -1.0)**	479	46.6%	
Ceftiofur (C)	780	30.7%	**-3.73 (-6.4 to -1.0)**	377	36.7%	**6.41 (2.2 to 11.0)**
Enrofloxacin (E)	231	10.4%	**+99.3 (30 to 205)**	---	---	
Florfenicol (F)	1226	48.3%	**-6.15 (-8.0 to -4.3)**	441	42.9%	3.99 (-1.2 to 9.4)
Gentamicin (G)	826	32.6%		---	---	
Neomycin (N)	978	38.5%		272	26.5%	-7.76 (-15.2 to +0.3)
Oxytetracycline (O)	2253	88.8%	**-1.35 (-2.2 to -0.5)**	547	53.2%	
Sulfadimethoxine (Sul)	2398	94.5%	-0.39 (-0.8 to +0.10)	850	82.7%	**-1.17 (-2.2 to -0.1)**
Trimethoprim/ Sulfamethoxazole (Ts)	838	33.0%		112	10.9%	

^a^ Spectinomycin was not included due to the availability of an accepted breakpoint for *Salmonella*

^b^ Annual rate of change in the proportion of resistant isolates for antimicrobials in which a significant trend (chi-square test for trends with p<0.05) was found.

**Fig 4:**
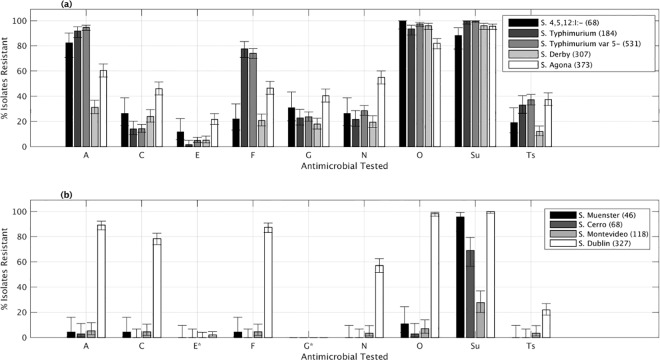
Relationship between serotype and antimicrobial resistance in *Salmonella* isolates recovered from swine and cattle at the MVDL, 2006–2015. Percentage of isolates recovered from swine (a) and cattle (b) samples resistant to ampicillin (A), ceftiofur (C), enrofloxacin (E), florfenicol (F), gentamicin (G), neomycin (N), oxytetracycline (O), spectinomycin (Sp), sulfadimethoxine (Sul), spectomycin (Sp) and trimethorpim/ sulfamethoxazole (Ts) belonging to the four most prevalent serotypes in each species plus *Salmonella* I 4,5,12:i:- serotype in swine per year.

Significant differences among serotypes were observed in the proportion of isolates resistant to A, C, E, F, G, O, N, Sul and Ts between the most prevalent serotypes Fig 4A). That finding was particularly evident for F (ranging from 21–22% in *S*. 4, 5, 12:i:- and *S*. Derby to ≥70% for *S*. Typhimurium and *S*. Typhimurium var 5-), A (from 31% in S. Derby to ≥80% in *S*. 4, 5, 12:i:-, Typhimurium and Typhimurium var 5-) and C (from 46% in *S*. Agona to ≤26% in the other more predominant serotypes) (Fig 4A). Although resistance to E was low in all serotypes, a significantly (Fisher’s Exact Test, p<0.05) higher proportion of resistant isolates were found among the *S*. Agona (21.7%) and *S*. 4,5,12:i:- (11.8%) serotype strains compared with *S*. Typhimurium (1.6%) and var 5- (5.1%).

### Cattle

**Fig 5:**
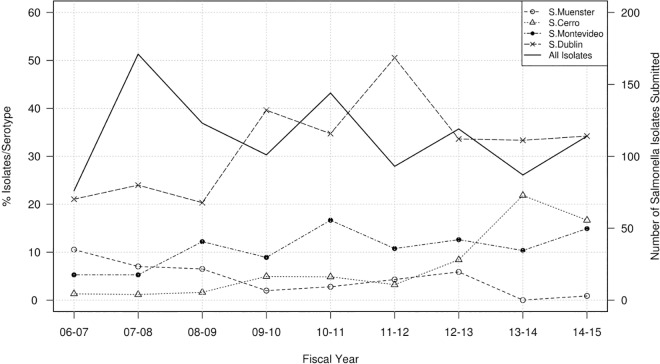
*Salmonella* serotypes recovered from cattle samples at the MVDL in 2006–2015. Number of isolates with a given serotype recovered from cattle samples per year (solid line, right axis) and percentage of these isolates belonging to the four predominant serotypes identified per year (dotted lines, left axis).

A total of 1,227 *Salmonella* isolates were characterized from bovine samples submitted from 880 farms in Minnesota. Those farms were reclassified in 1,114 farm/month-years, of which 151 (13.6%) were excluded due to the lack of data on resistance to the antibiotics of interest. Exclusion of duplicate strains recovered from the same farm and month-year led to 1,028 cattle isolates included in the analysis. The average number of isolates recovered at the MVDL per fiscal year was 114 (range 76–171), with no significant trend over time (Fig 5). Tissue samples (liver, intestine, lung, spleen and lymph nodes primarily) were again the main sample types from which *Salmonella* were recovered (50% of all isolates), whereas 38% of the cattle isolates were cultured from fecal samples.

The 1,028 isolates were classified into 60 different serotypes, except 12 isolates classified as untypable or containing multiple serotypes (S2 Table). The four most prevalent bovine serotypes (accounting for >50% of the serotyped isolates) were *S*. Dublin (n = 327 isolates, 31.8%), *S*. Montevideo (n = 118, 10.9%), *S*. Cerro (n = 68, 6.6%) and *S*. Muenster (n = 46, 4.5%) (Fig 5). A significant (p<0.001) decreasing trend was found for *S*. Muenster, which was the second most common serotype in 2006–2007 but was found only once in 2014–2015, while a significant (p<0.001) increasing trend was found for the other three most prevalent serotypes (Fig 5).

MICs recorded each year were also significantly different (Kruskal-Wallis test, p<0.001) for all antimicrobials evaluated in cattle, except C. Proportion of cattle *Salmonella* isolates belonging to each MIC over the study period can be observed in S2 Fig. Here, the cumulative logit regression analysis revealed the existence of significant trends for N, O and Sp (decreasing resistance), F (increasing resistance) and C (increasing probability of having MICs ≤0.5 μg/ml and decreasing of having MICs ≥1 μg/ml (Table 2).

**Fig 6:**
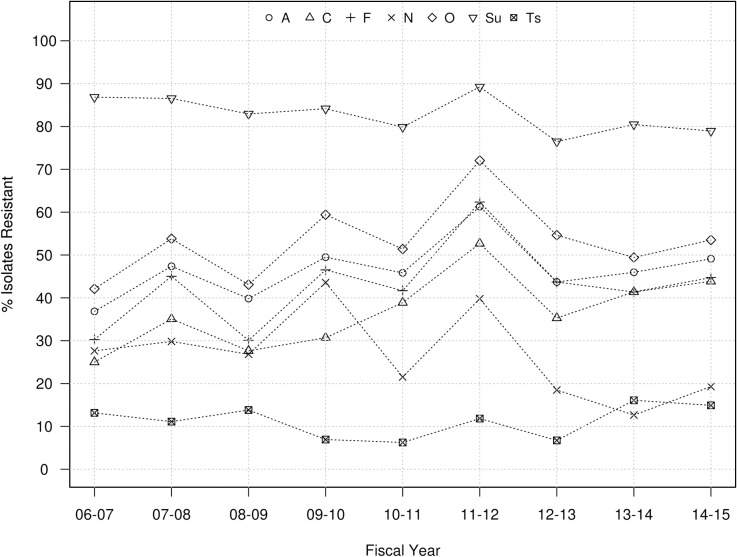
Evolution in antimicrobial resistant *Salmonella* isolates recovered from cattle at the MVDL in 2006–2015. Proportion of *Salmonella* isolates recovered from cattle samples that were resistant to ampicillin (A), ceftiofur (C), florfenicol (F), neomycin (N), oxytetracycline (O), sulfadimethoxine (Su), spectomycin (Sp) and trimethorpim/sulfamethoxazole (Ts).

Proportion of resistant isolates was high (76–87%) for Sul, variable for A, C, F and O (between 30 and 70%), and low for N and particularly Ts (<15%) across years (Fig 6). No significant 9-year trends were seen for A, F, N, O, and Ts. In contrast, significant decreasing (Sul) and increasing (C) trends in the proportion of resistant isolates were detected, with more abrupt changes between years compared to swine (Table 3 and Fig 6). A total of 56 isolates (4%) were resistant to all 7 antimicrobials, and the overall median number of resistances per isolate was 3, though it ranged between 1 and 5 depending on the year (Fig 3).

When only antimicrobials tested in all strains regardless their host species (n = 7) were considered, a significantly (Mann-Whitney test, p<0.001) lower number of resistances was found in cattle isolates (median number = 3) compared with swine (median number = 4). That difference increased if the cattle *S*. Dublin isolates were excluded, with the median number of resistance in cattle isolates dropping to 1 (Fig 3). The proportions of isolates resistant to A, C, F, N, O, and Ts were significantly higher for *S*. Dublin compared to other serotypes (*p* <0.001) (Fig 4B). The most prevalent resistotype (R3) in cattle was Sul, found in 29% of the cattle isolates (198/1028), followed by resistotype A-C-F-O-Sul (n-136, 13%). Additionally, the R2 profile was also most common among isolates of serotypes *S*. Typhimurium (7/26) and Typhimurium var. 5- (8/36).

### Resistotype diversity per host species

**Fig 7:**
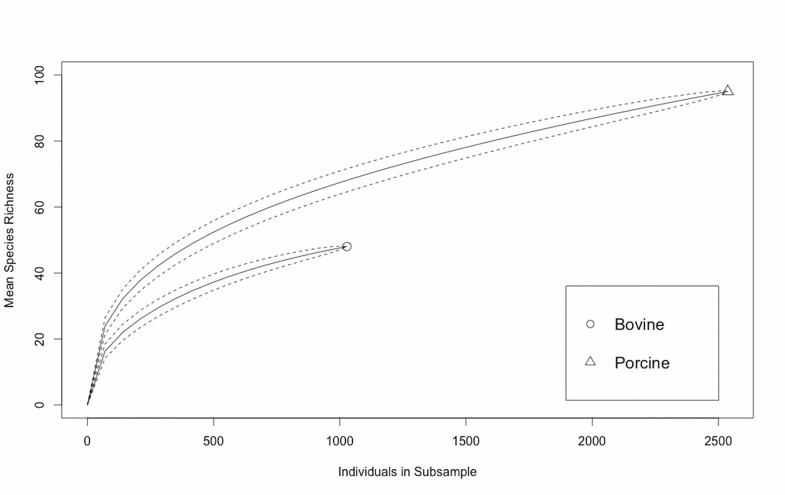
Resistotype richness in *Salmonella* isolates recovered from swine and cattle at the MVDL in 2006–2015. Sample-based rarefaction curves comparing the estimated mean resistotype richness in porcine and bovine samples from Minnesota at different sampling efforts (95% confidence intervals are indicated by a dotted line).

Rarefaction was performed to compare the number of resistotypes as a function of the number of samples per source. Richness of resistotypes was larger in porcine than bovine isolates (Fig 7), and these results were consistent if sub-divided into individual years (data not shown). Simpson’s index of diversity of bovine and porcine resistotypes was 0.86 (95% confidence interval: 0.85–0.87) and 0.93 (95% confidence interval: 0.93–0.94), respectively.

The PSI between resistotypes in bovine and porcine isolates was 0.42 with a bootstrapped 95% confidence interval of 0.39–0.44. The PSI of resistotypes in the four most common serotypes (representing 55 and 54% of porcine and bovine isolates, respectively) within each source is presented in Table 4. In porcine, *S*. Typhimurium and *S*. Typhimurium var 5- isolates showed a significantly higher similarity than all other comparisons. For bovine, low similarity was detected in all comparisons with the exception of bovine *S*. Cerro isolates, which appeared to have a significantly higher similarity with both *S*. Montevideo and *S*. Muenster.

**Table 4:** Proportional similarity index (with bootstrapped 95% confidence intervals) of the top four bovine and top four porcine serotypes based on resistance to the 13 core antimicrobials.

		**Bovine**		
**Serotype**	***S*. Cerro**	***S*. Dublin**	***S*. Montevideo**	***S*. Muenster**
***S*. Cerro**	-			
***S*. Dublin**	0 (0, 0)	-		
***S*. Montevideo**	0.36 (0.24, 0.45)	0.04 (0.01, 0.08)	-	
***S*. Muenster**	0.06 (0.01, 0.12)	0.03 (0, 0.07)	0.21 (0.13, 0.29)	-
Number of isolates	69	369	118	53
		**Porcine**		
**Serotype**	***S*. Agona**	***S*. Derby**	***S*. Typhimurium**	***S*. Typhimurium var 5-**
***S*. Agona**	-			
***S*. Derby**	0.42 (0.33, 0.44)	-		
***S*. Typhimurium**	0.20 (0.13, 0.23)	0.17 (0.11, 0.19)	**-**	
***S*. Typhimurium var 5-**	0.23 (0.18, 0.26)	0.18 (0.14, 0.21)	0.80 (0.70, 0.81)	**-**
Number of isolates	380	311	184	536

## Discussion

Among foodborne pathogens in the USA, *Salmonella* infections are estimated to cause the greatest societal burden in terms of both overall economic cost and attributable deaths [30, 31]. Furthermore, compared with antibiotic susceptible *Salmonella*, presence of multiple antibiotic resistances has been associated with increased morbidity, mortality, risk of bloodstream infections and hospitalization rates in infected humans. [32, 33]. Although the incidence of clinical *Salmonella* cases in people has remained relatively stable over the last 20 years, it is encouraging that national surveillance data have shown a substantial decline in the proportion of clinical isolates with multiple antibiotic resistant phenotypes [34]. The relatively high prevalence of multiple antibiotic resistances observed here is consistent with data from NARMS indicating that *Salmonella* isolates from animal carcasses at harvest and retail meats are more frequently multiple resistant than human clinical isolates [35], even if a large proportion of them are pansusceptible.

Comparison of findings obtained in different studies/laboratories should be viewed with caution, given that many (often unobserved) factors may affect results, such as different isolation and culture methods (when findings from different laboratories are compared), animal demographics and management practices, environmental conditions, antimicrobial uses, and presence of other pathogens (when results from populations in different geographical areas are compared) [12, 15]. To account for the influence of these factors, standardized laboratory procedures from the MVDL were done on animals from a common geographic area (i.e. Minnesota), what could help to minimize part of that variability, although changes in the animal populations sampled throughout the study period may have contributed to part of the observed changes in serotype distributions/AMR prevalence.

The number of isolates recovered from both swine and cattle declined over the study period (Figs 1 and 5), on average, 286 and 136 isolates belonging to different serotypes and/or with different resistotypes were isolated each year from different farms (>1,800 swine farms and >800 cattle herds overall) located in Minnesota, thus offering a valuable picture of the most common serotypes and antimicrobial resistance profiles present in isolates recovered from clinically affected animals. Isolates were derived from clinical submissions but clinical and diagnostic information was not available, and ultimately the role of *Salmonella* as the cause of illness is not readily apparent. This lack of information on clinical significance of the isolate may be particularly important for cases in which *Salmonella* was only recovered through an enrichment step (in which bacterial load was likely lower). No significant differences in the proportion of isolate source by serotype were observed in swine, whereas *S*. Dublin was more commonly recovered from tissue samples. *S*. Dublin isolates were recovered from liver (87%), lung (90%), intestine (43%) and fecal (4%) samples, reinforcing the clinical relevance of *S*. Dublin as a cattle pathogen [36].

A hallmark of *Salmonella* epidemiology is the variability of serotype prevalence over time. It has extensively been observed, in human and animal populations, that particular serotypes and phage types may abruptly increase or decrease, but the causal mechanisms underlying those changes are poorly understood [37, 38]. This was evident in our study as well as other in previous national studies of US swine and cattle [14, 39]. Interestingly, among the most common serotypes found in swine through the NARMS slaughterhouse sampling between 2006 and 2013 (Derby, Typhimurium, Anatum, Johannesburg and Infantis) [10], only *S*. Derby and *S*. Typhimurium were among the top four serotypes in the MVDL collection (S1 Table). It was observed from the MVDL swine isolates a decreasing trend in the frequency of *S*. Derby and *S*. Typhimurium from swine samples which was also observed in the NARMS data from 1997 until 2011 [39] (Fig 1). However, there was a relatively small number of swine isolates serotyped as part of the NARMS project in several of years (<150 isolates/year in 2008–2011) limiting the comparison between both datasets. In contrast, the prevalence of *S*. Agona increased and, to a lesser extent, *S*. 4,5,12:i:- in swine in the MVDL samples, but this was not observed in the NARMS data. This is of particular interest for serotype 4,5,12:i:- given its emergence as an important cause of foodborne salmonellosis in the U.S. and elsewhere [39, 40–42], and that it has caused outbreaks associated with consumption of pork products [43, 44]. In addition, over 90% of the *S*. Agona isolates recovered were from swine samples, whereas in the 1997–2003 NARMS data, cattle were the predominant source of *S*. *agona* in slaughter samples from food animals [13]. However, differences in the frequency of different serotypes in swine between our dataset and the NARMS were not unexpected given the different populations sampled (healthy animals at the slaughterhouse versus clinically affected animals at the farm). In contrast, a close agreement between both sources of isolates was found for cattle, with serotypes Dublin and Montevideo ranking as the most common serotypes in both studies. However, the host-adapted *S*. Dublin was by far the most common serotype among the MVDL diagnostic samples, whereas *S*. Montevideo was consistently the predominant serotype in the NARMS cattle data from 2006 to 2013 [10] (Fig 5).

Comparison of our AMR results with those from NARMS studies is limited by the observation that only four antibiotics tested at the MVDL (A, C, G and Ts) were also tested in NARMS. However, those assessments are clinically relevant because ampicillin and trimethoprim-sulfamethoxazole are among the traditional options for treatment of invasive *Salmonella* infections, and ceftiofur, a 3^rd^ generation Cephalosporin, is related to ceftriaxone, used to treat *Salmonella* infection in children [45, 46]. Among the MVDL swine isolates, the proportion of resistant strains to those four antimicrobials was higher compared with the NARMS data (even if only NARMS clinical isolates cultured between 1997 and 2006 are considered) [39] and with isolates from diseased animals collected at other state veterinary diagnostic laboratories [47]. Still, the limited but significant decreasing trend observed in the MICs and the proportion of resistant isolates against A and C recorded over the years suggest that in the last 10 years the level of resistance to these two antimicrobials has been decreasing consistently.

For cattle, the proportion of resistant isolates were similar between the MVDL clinical isolates and the NARMS and clinical veterinary diagnostic isolate collections [10, 47] for the three antibiotics tested in common. Notably, proportion of isolates resistant to C increased markedly (from 0.5 to 40%) among clinical NARMS isolates between 1997 and 2006, and the MVDL data indicate this was continued or increased in subsequent years (going from 38% in 2006 to 46% in 2015) (Fig 6), though no trends were observed at the MIC level. Proportion of ceftiofur-resistant isolates among cattle isolates (37.4%) was higher than that observed in swine strains (30.6%) (Table 3), as previously described for *Salmonella* isolates recovered from clinical and slaughterhouse samples from both animal species [48, 49].

Comparisons between MVDL data and those described in the NARMS clinical samples must consider the different timeframes in which the strain collections were formed. That potential bias may be avoided by using data from the NARMS isolates recovered through slaughterhouse sampling (available until 2013), in which more extreme differences are found for both cattle and swine isolates, probably due to the different populations considered (clinically affected versus healthy), as observed also when NARMS data from both sources (slaughterhouse and clinical samples) were compared [48, 50]. However, care must be taken when comparing trends of resistance to antimicrobials considered separately (as performed here or in the NARMS reports), since simultaneous resistance to other antibiotics may have a major effect on these trends over time [12].

As previously observed [47–49, 51], the proportion of isolates resistant to respective antimicrobials varied among serotypes for both swine and cattle isolates (Fig 4). In swine, no single serotype was particularly resistant to all the antimicrobials included in the panel. However, *S*. Typhimurium and *S*. Agona isolates were more resistant than isolates from other serotypes, in agreement with previous reports [47–49, 51]. In cattle, *S*. Dublin was the most resistant serotype (Fig 4), also in agreement with a previous study [47]. That finding was also supported by the low PSI obtained in this serotype compared with the other three predominant serotypes in cattle (Table 4).

A possible explanation for some of the temporal changes in serotype and AMR prevalence could include a systematic bias derived from the potential variability on the sampled population due to changes in the swine and cattle farms submitting samples to the MVDL. However, the submitting population was highly variable, so that no more than 6% of the farms from which *Salmonella* isolates were recovered in a given year were present in any other year. Hence, no systematic bias associated with specific farms submitting samples on certain periods is expected. To control for this potential effect, for farms submitting multiple samples in a short period of time we included one single isolate per combination of serotype-resistotype recovered from a given farm in a given month and year. This was similar to what was done before in a previous study [52] and likely decreases the bias associated with oversampling at the farm level, since there were no farms accounting for more than 0.5% of all isolates for either animal species.

The increasing MIC levels for E (reflected also in the proportion of E-resistant isolates) in *Salmonella* strains recovered from swine was interesting, given the previous lack of susceptibility information for this antimicrobial, that was licensed for use in swine in 2008 in the US [53], and could be related in part with a shift in the predominant serotypes, since also in two of the most frequent in the last years (*S*. Agona and *S*. 4,5,12:i:-) an increased proportion of resistant isolates against this antimicrobial were found (Fig 4). Further monitoring of fluoroquinolone susceptibility is needed prospectively.

Although there is a general agreement that antimicrobial use in animals select for AMR the degree of its impact is not clear [54, 55]. In the present study we have demonstrated that overall isolates recovered from swine were resistant to a greater number of antimicrobials than those coming from cattle (Fig 3). The rarefaction analysis illustrated a larger resistotype richness among porcine isolates compared to bovine isolates, though diversity of resistotypes was high in both sources. However, rarefaction analysis cannot be used to assess if the target population has been sampled sufficiently as it can only provide information on the sampled population and hence cannot be used to assess representativeness of the resistotypes identified. Regardless, richness is expected to increase with sample size; therefore, the expected number of resistotypes given the same number of samples can be estimated using rarefaction [56]. Further, the shapes of the rarefaction curves were used to determine if additional samples would likely detect new resistotypes from each source in alignment with the approach taken by Muellner et al. [57]. The porcine isolates had also a slightly, but significantly, higher Simpson’s Index of diversity than bovine isolates. The AMR diversity observed in our samples was considerably higher than in previous work from Canada, where samples collected from asymptomatic swine had a Simpson’s Index for AMR in *S*. Typhimurium DT104 between herds of 0.77 and within herds of 0.27 [58]. Also, a higher proportion of resistant swine isolates was found compared with diagnostic isolates recovered through NARMS in 1997–2006 for ampicillin, ceftiofur, gentamicin and trimethoprim-sulfamethoxazole, while this was not the case for cattle. Results from the PSI analysis indicate some serotypes are more similar in their resistotype profile than others (particularly *S*. Typhimurium and var 5- and to a lesser extent *S*. Agona and Derby in swine, and *S*. Montevideo and Cerro in cattle) (Table 4). Further genomic exploration is required to investigate this; however the diversity, richness and similarity analysis has provided some additional insight into the nature of AMR in the two sources investigated. The spatial (or temporal) distribution of samples included here may not be assumed to be homogeneous, what could thus potentially influence the observed results if for example samples from a certain area in which certain epidemiological conditions influenced *Salmonella* circulation were submitted only for a given period of time, what could lead to changes in the overall trends due to a spatio-temporal restricted phenomenon. However, if potential biases may be assumed constant through time (so that sampling biases are maintained and therefore changes found reflect a true change in the sampled population–clinically affected animals), methods here show the potential for use of data routinely collected at veterinary diagnostic laboratories in monitoring prevalence of AMR in food animal species, what could help in the design, implementation and monitoring of policy intended to prevent or mitigate AMR impact on animal health in the U.S. Comparison with trends observed in human clinical isolates throughout the same period may allow the detection of similar trends (i.e., increase in certain serotypes and/or AMR patterns) that could be useful to clarify links between human and animal health, though careful interpretation of these findings would be required given the biased population (clinically affected animals) represented in this kind of animal databases.

## Supporting Information

S1 Fig MICs in *Salmonella* isolates recovered from swine per year in 2006–2015. Distribution of the proportion of Salmonella isolates recovered from swine showing each minimum inhibitory concentration (MIC) per year: 06–07, n = 352; 07–08, n = 384; 08–09, n = 283; 09–10, n = 235; 10–11, n = 309; 11–12, n = 307; 12–13, n = 247; 13–14, n = 240; 14–15, n = 180 except for enrofloxacin in 07–08, n = 67).
(DOCX)

S2 Fig MICs in *Salmonella* isolates recovered from cattle per year in 2006–2015. Distribution of the proportion of *Salmonella* isolates recovered from cattle showing each minimum inhibitory concentration (MIC) per year: 06–07, n = 76; 07–08, n = 171; 08–09, n = 123; 09–10, n = 101; 10–11, n = 144; 11–12, n = 93; 12–13, n = 119; 13–14, n = 87; 14–15, n = 114.
(DOCX)

S1 Table Number of isolates (N) belonging to each serotype recovered from swine samples at the MVDL in 2006–2015. (CSV)

S2 Table Number of isolates (N) belonging to each serotype recovered from cattle samples at the MVDL in 2006–2015. (CSV)
