# Supplementary material for: Serotypes and Antimicrobial Resistance in Salmonella enterica Recovered from Clinical Samples from Cattle and Swine in Minnesota, 2006 to 2015
Source: PLoS One. 2016 Dec 9;11(12):e0168016. doi: 10.1371/journal.pone.0168016 (PMC5148076; doi:10.1371/journal.pone.0168016)

**S1 Fig. MICs in Salmonella isolates recovered from swine per year in 2006-2015**Distribution of the proportion of Salmonella isolates recovered from swine showing each minimum inhibitory concentration (MIC) per year: 06-07, n=352; 07-08, n=384; 08-09, n=283; 09-10, n=235; 10-11, n=309; 11-12, n=307; 12-13, n=247; 13-14, n=240; 14-15, n=180 except for enrofloxacin in 07-08, n=67).


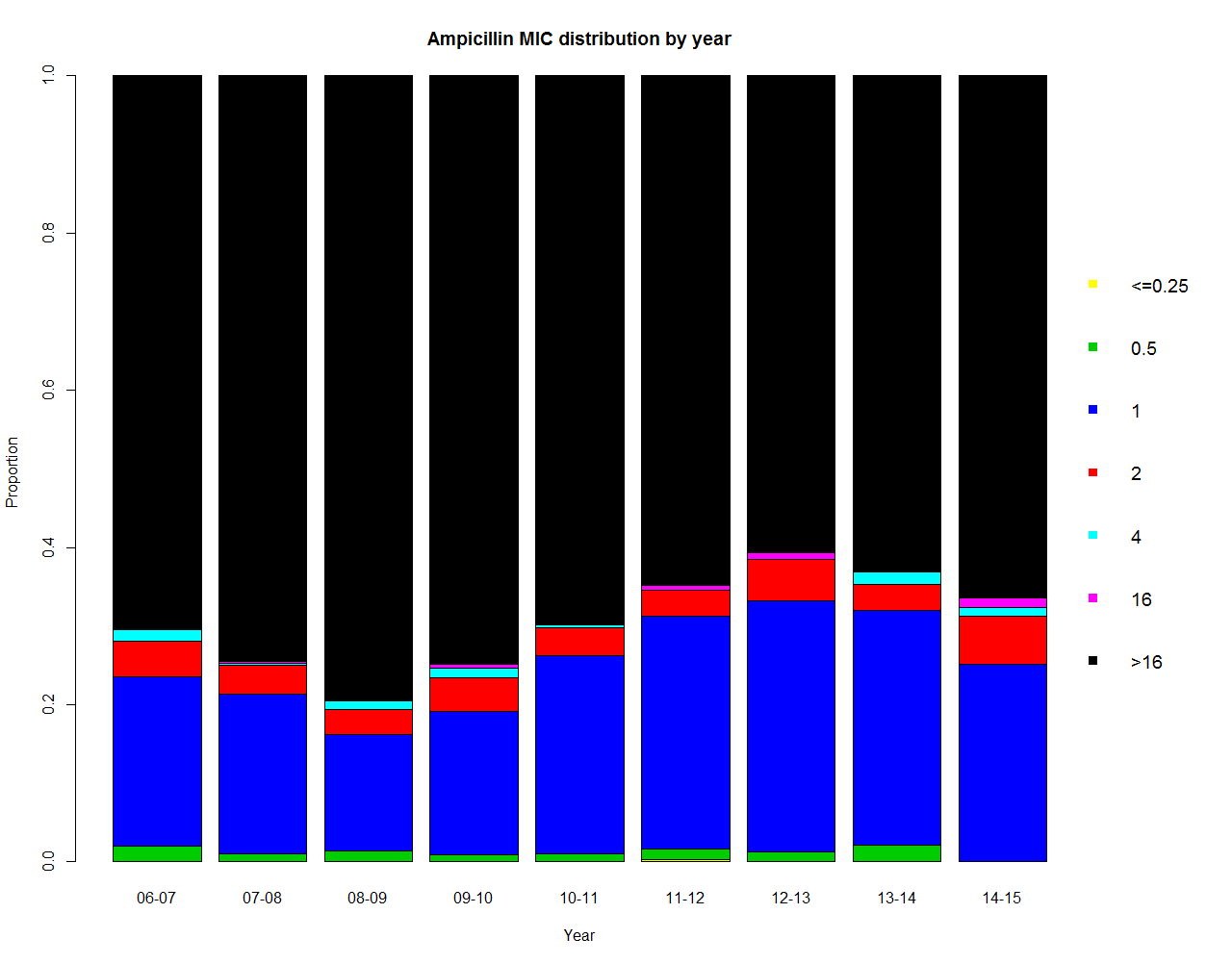


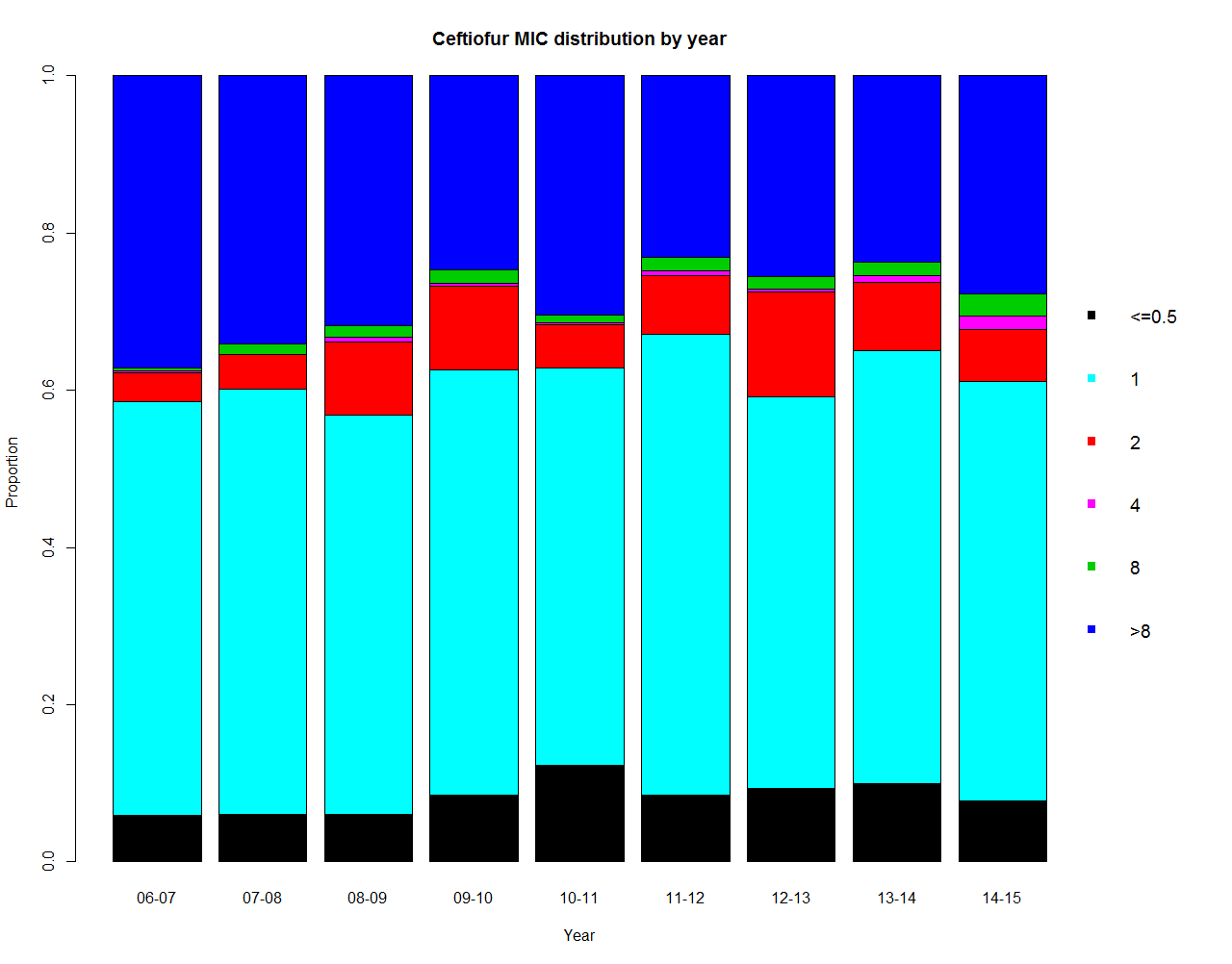


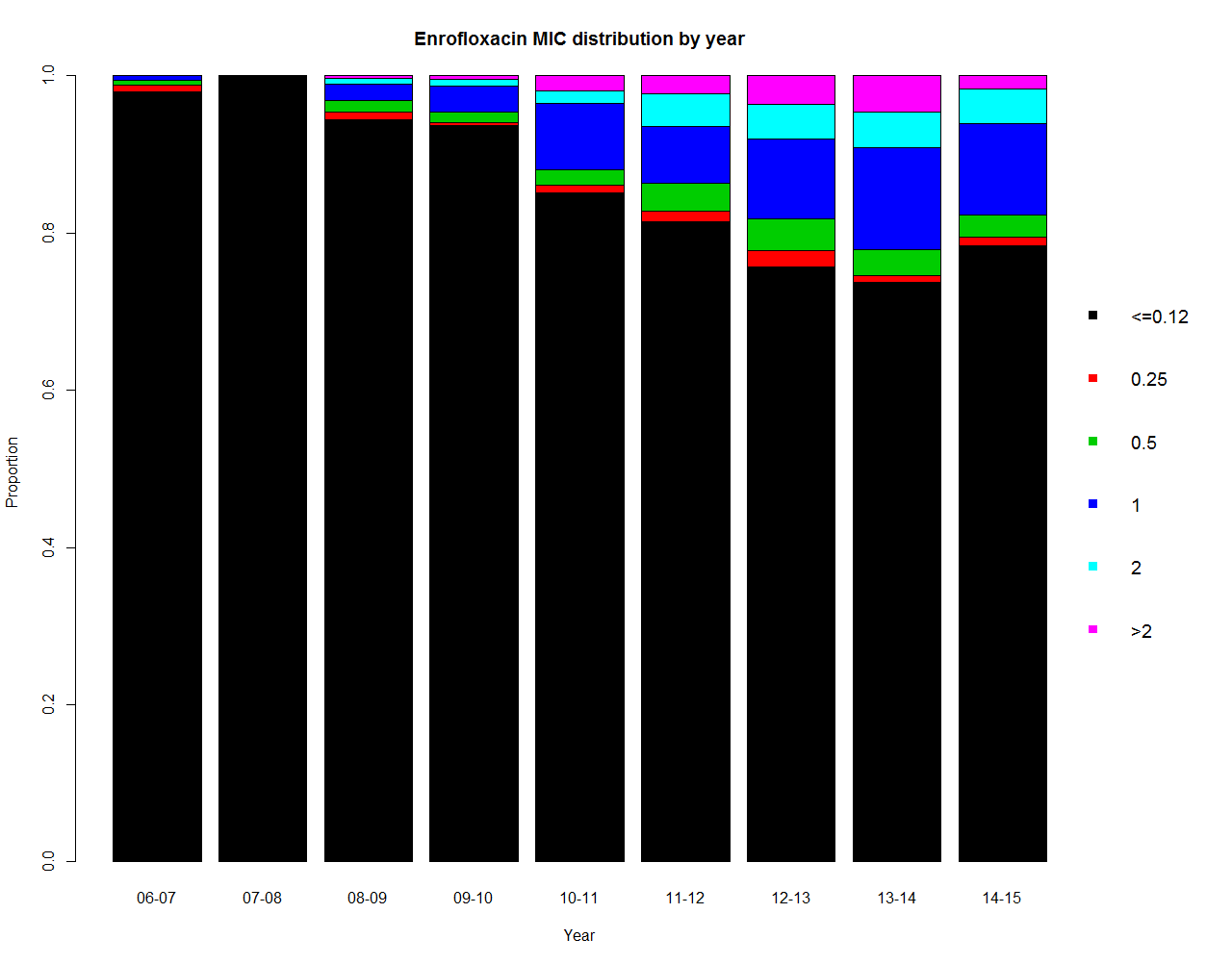

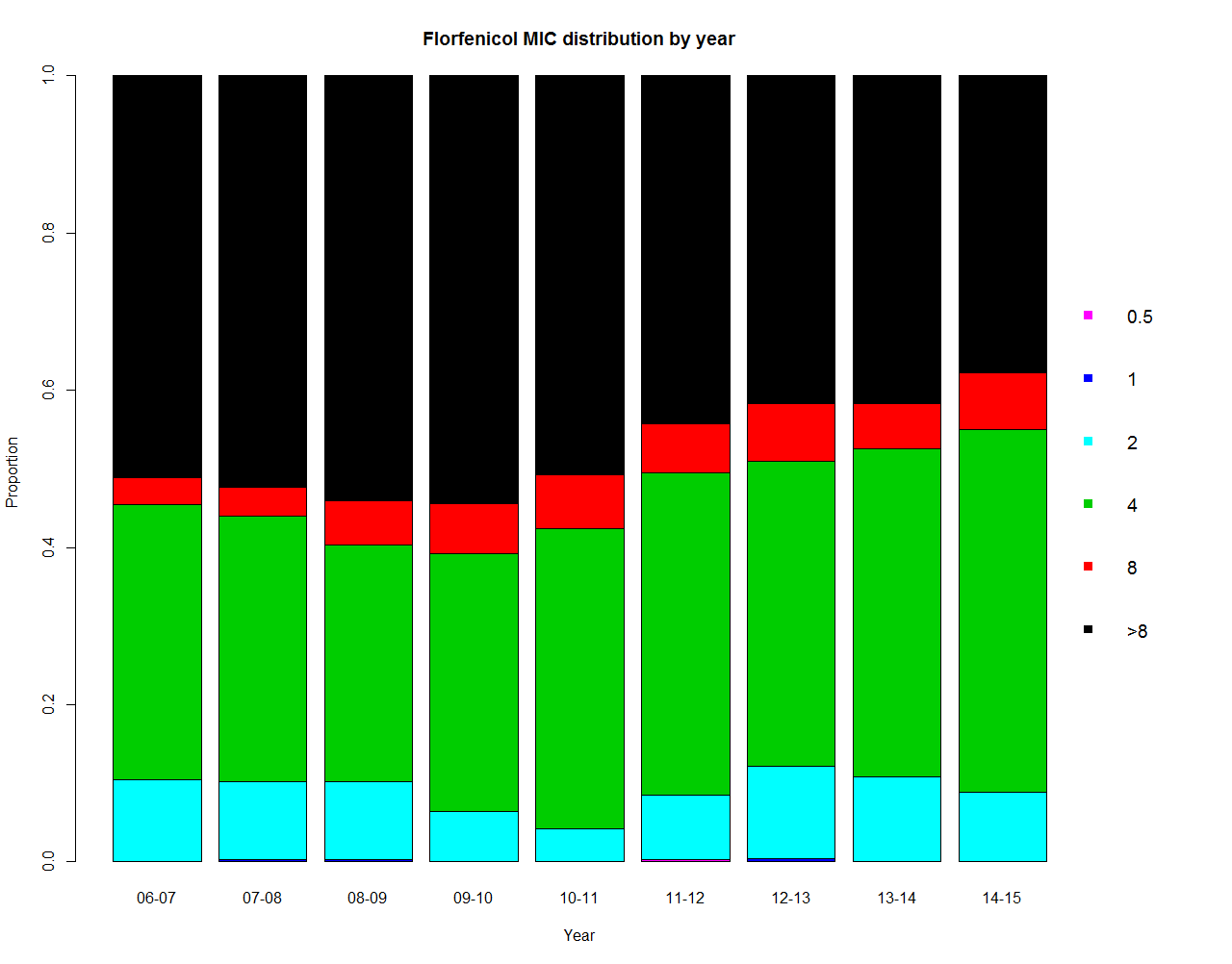

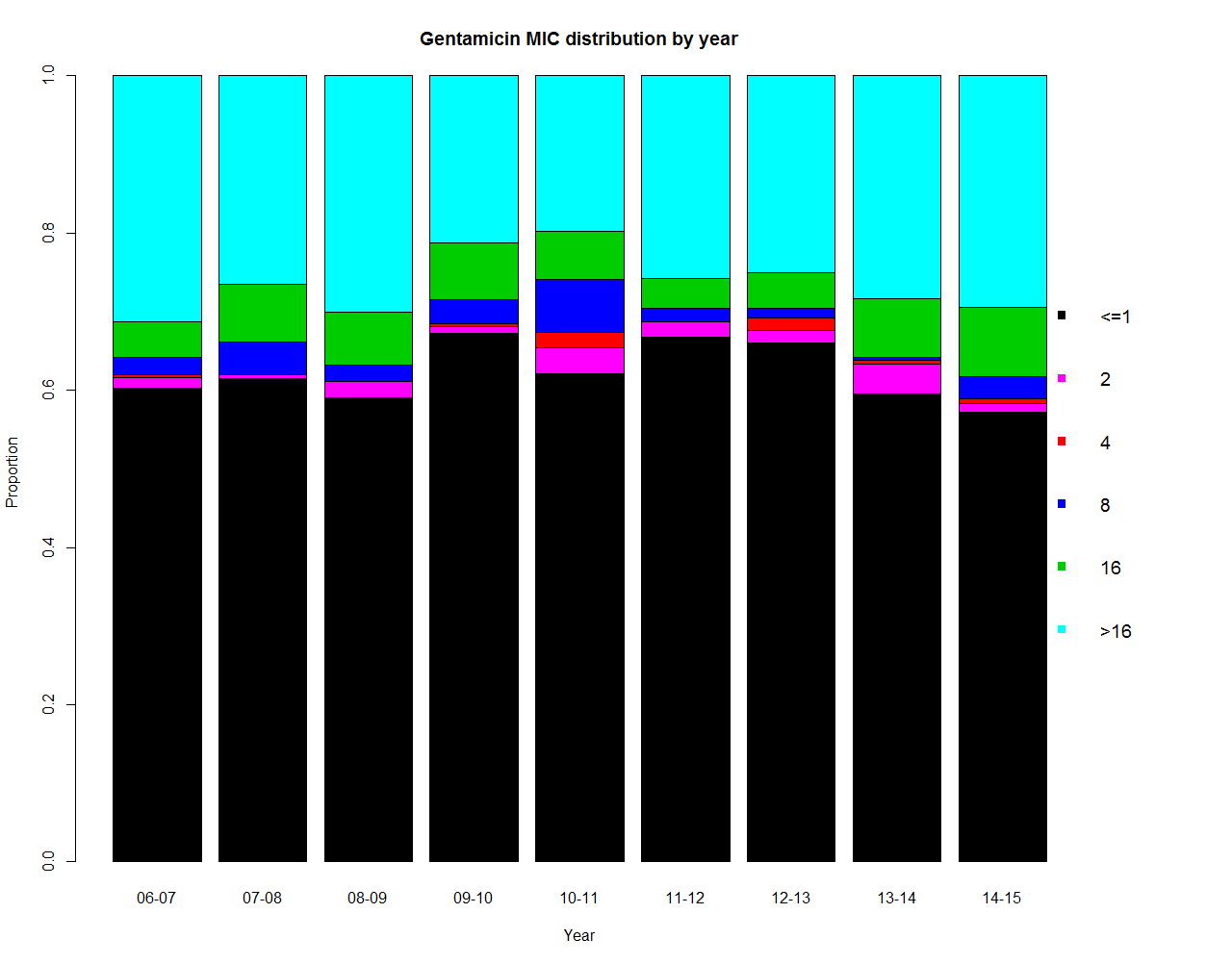

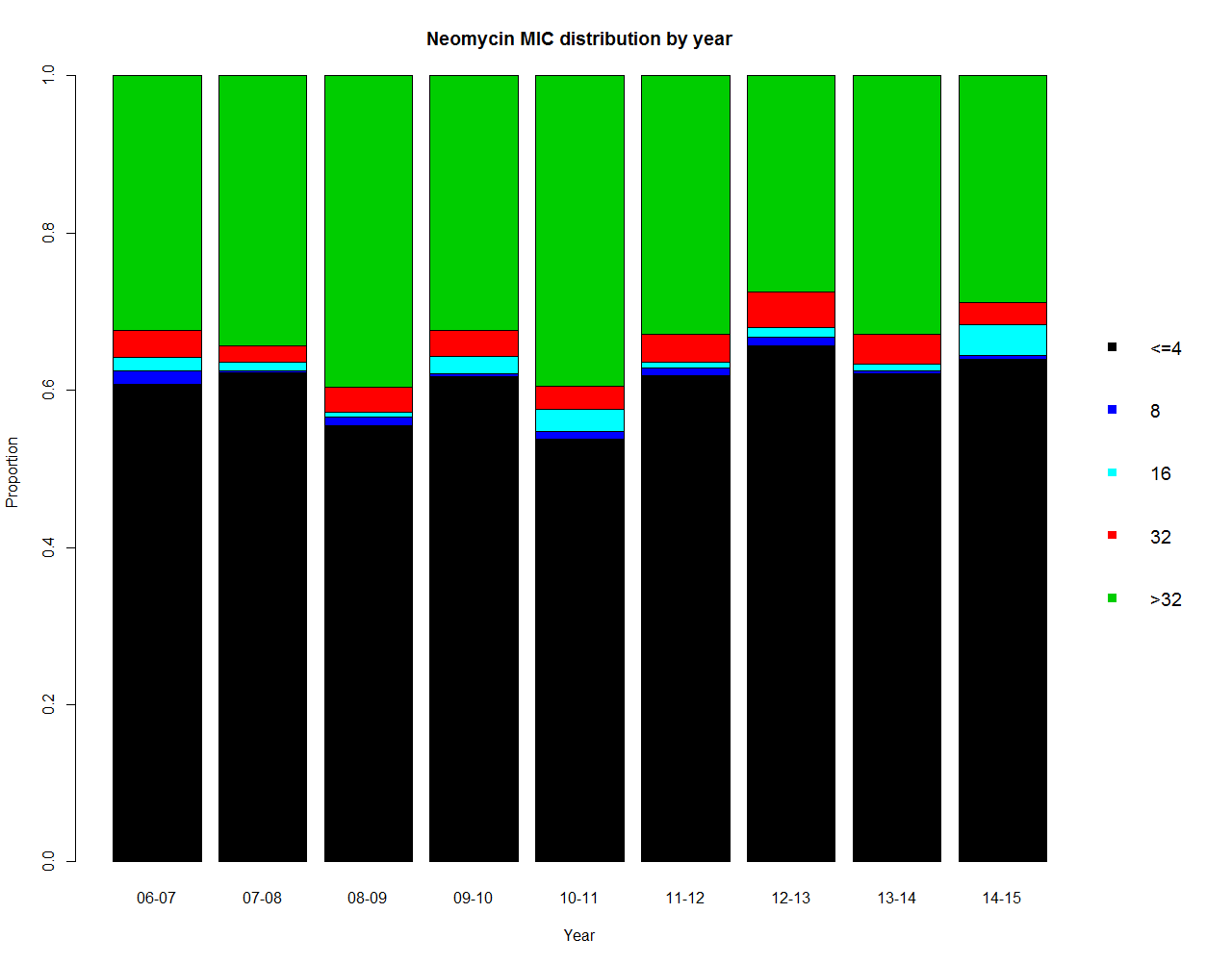

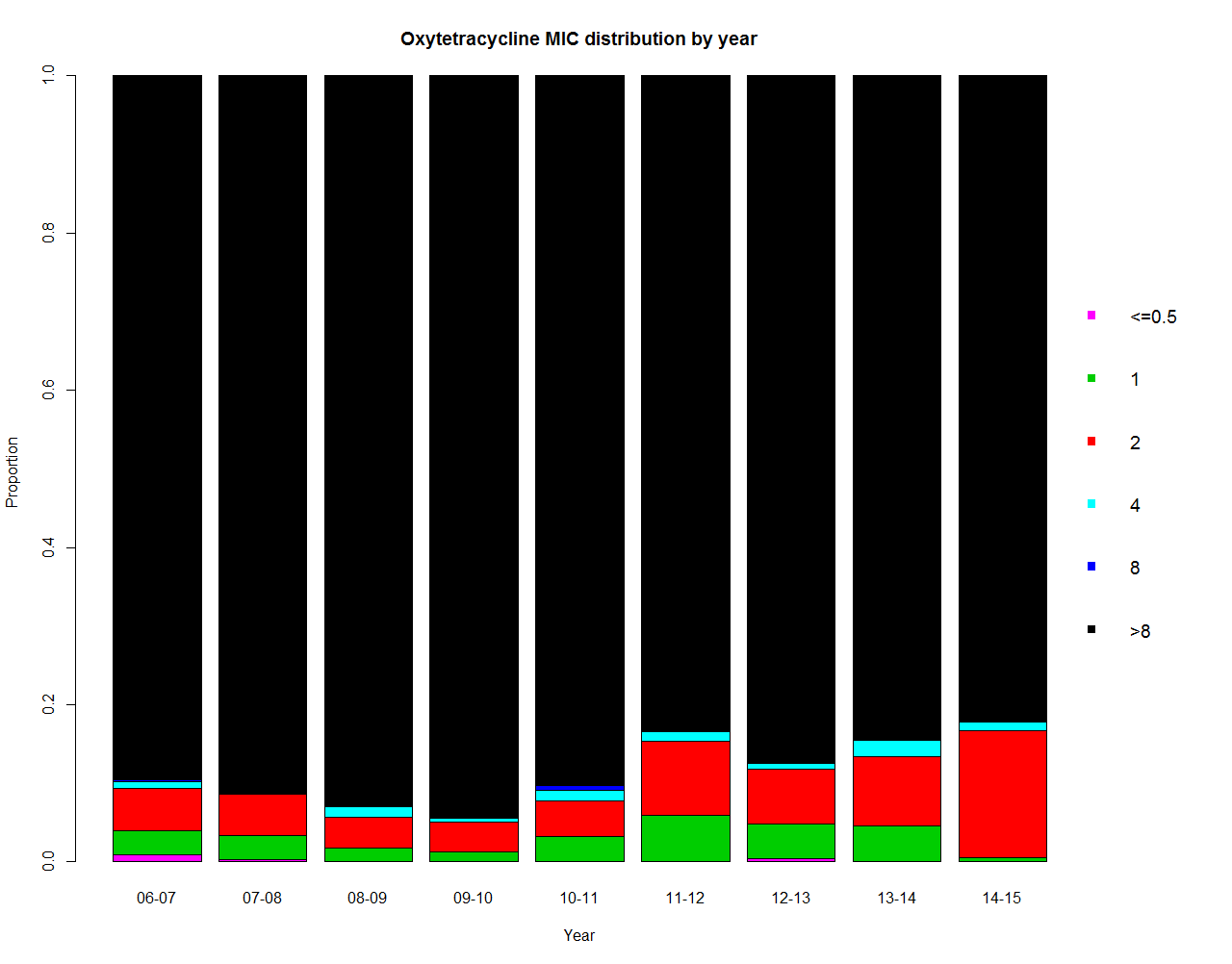


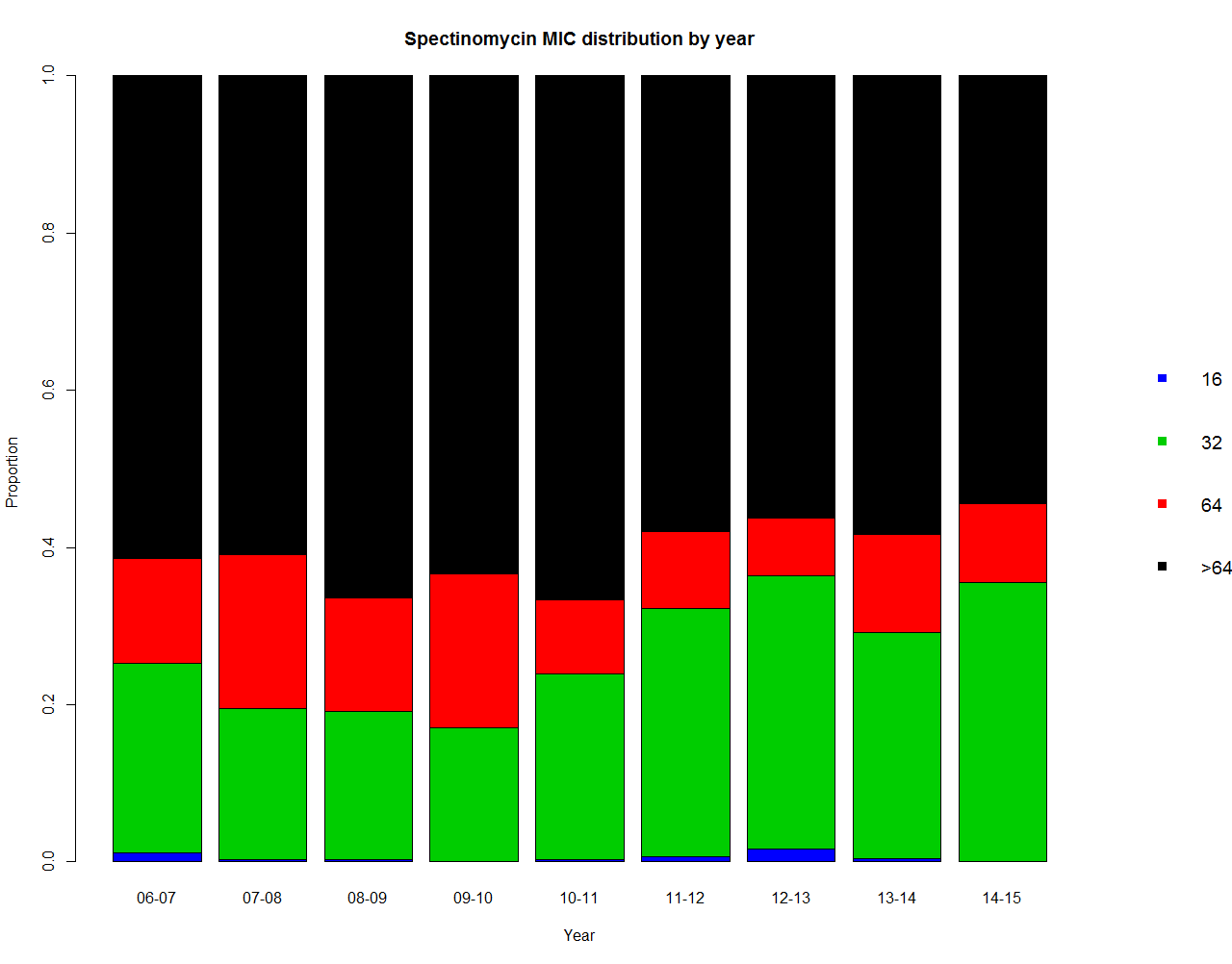

Supplement: S1 Fig — Distribution of the proportion of Salmonella isolates recovered from swine showing each minimum inhibitory concentration (MIC) per year: 06–07, n = 352; 07–08, n = 384; 08–09, n = 283; 09–10, n = 235; 10–11, n = 309; 11–12, n = 307; 12–13, n = 247; 13–14, n = 240; 14–15, n = 180 except for enrofloxacin in 07–08, n = 67). (DOCX) [file pone.0168016.s001.docx]
