# Supplementary material for: Serotypes and Antimicrobial Resistance in Salmonella enterica Recovered from Clinical Samples from Cattle and Swine in Minnesota, 2006 to 2015
Source: PLoS One. 2016 Dec 9;11(12):e0168016. doi: 10.1371/journal.pone.0168016 (PMC5148076; doi:10.1371/journal.pone.0168016)

**S2 Fig. MICs in *Salmonella* isolates recovered from cattle per year in 2006-2015.** Distribution of the proportion of *Salmonella* isolates recovered from cattle showing each minimum inhibitory concentration (MIC) per year: 06-07, n=76; 07-08, n=171; 08-09, n=123; 09-10, n=101; 10-11, n=144; 11-12, n=93; 12-13, n=119; 13-14, n=87; 14-15, n=114.
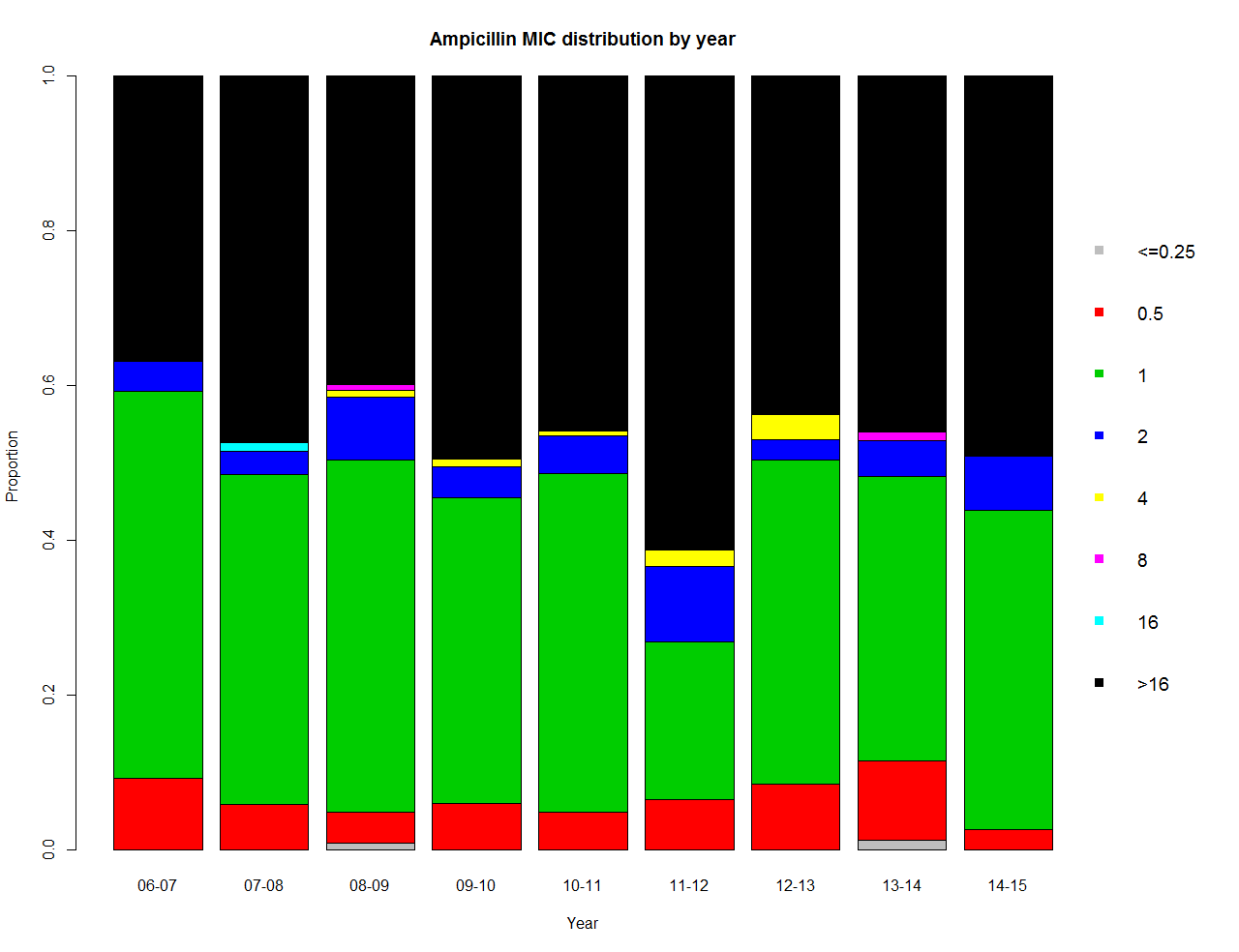

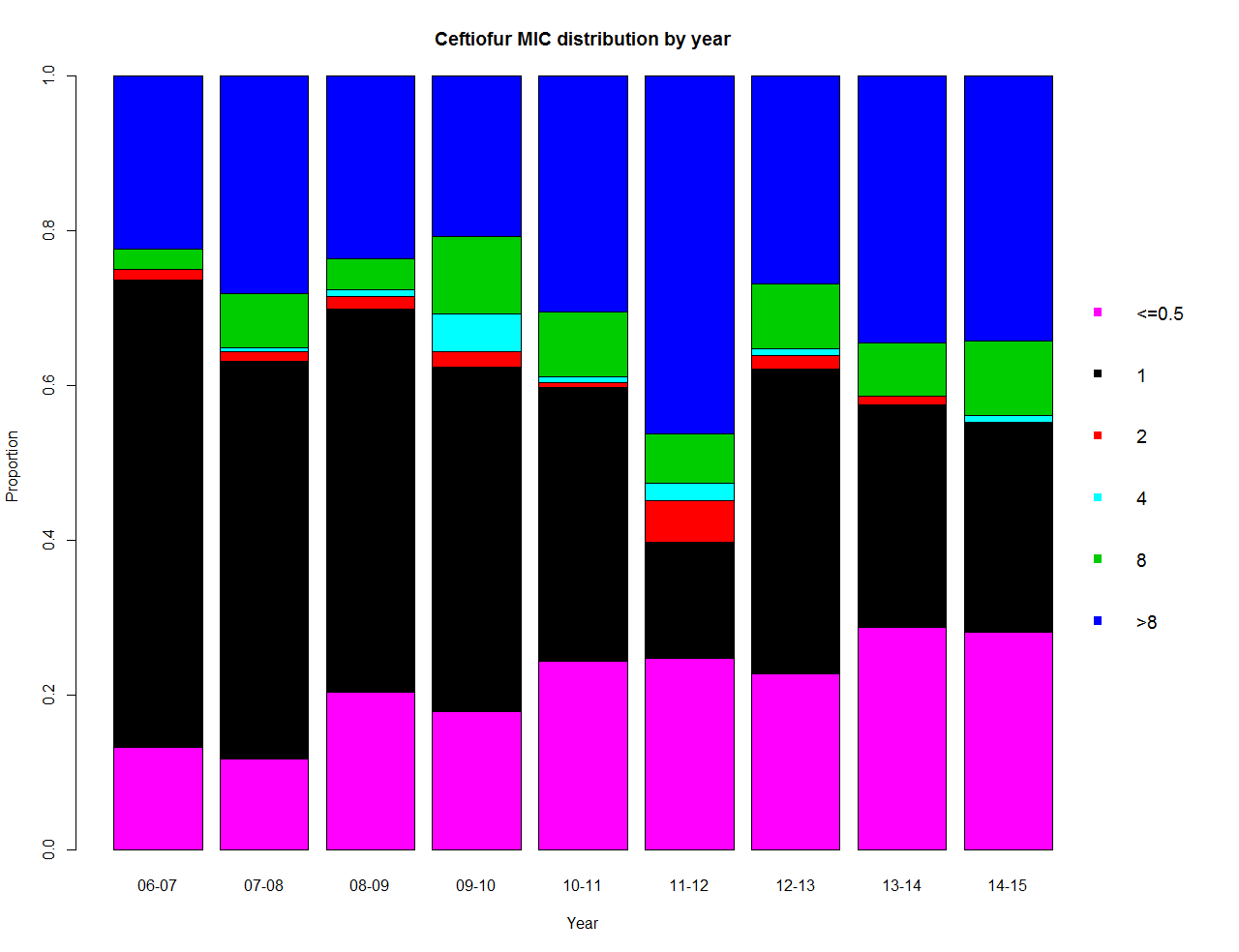

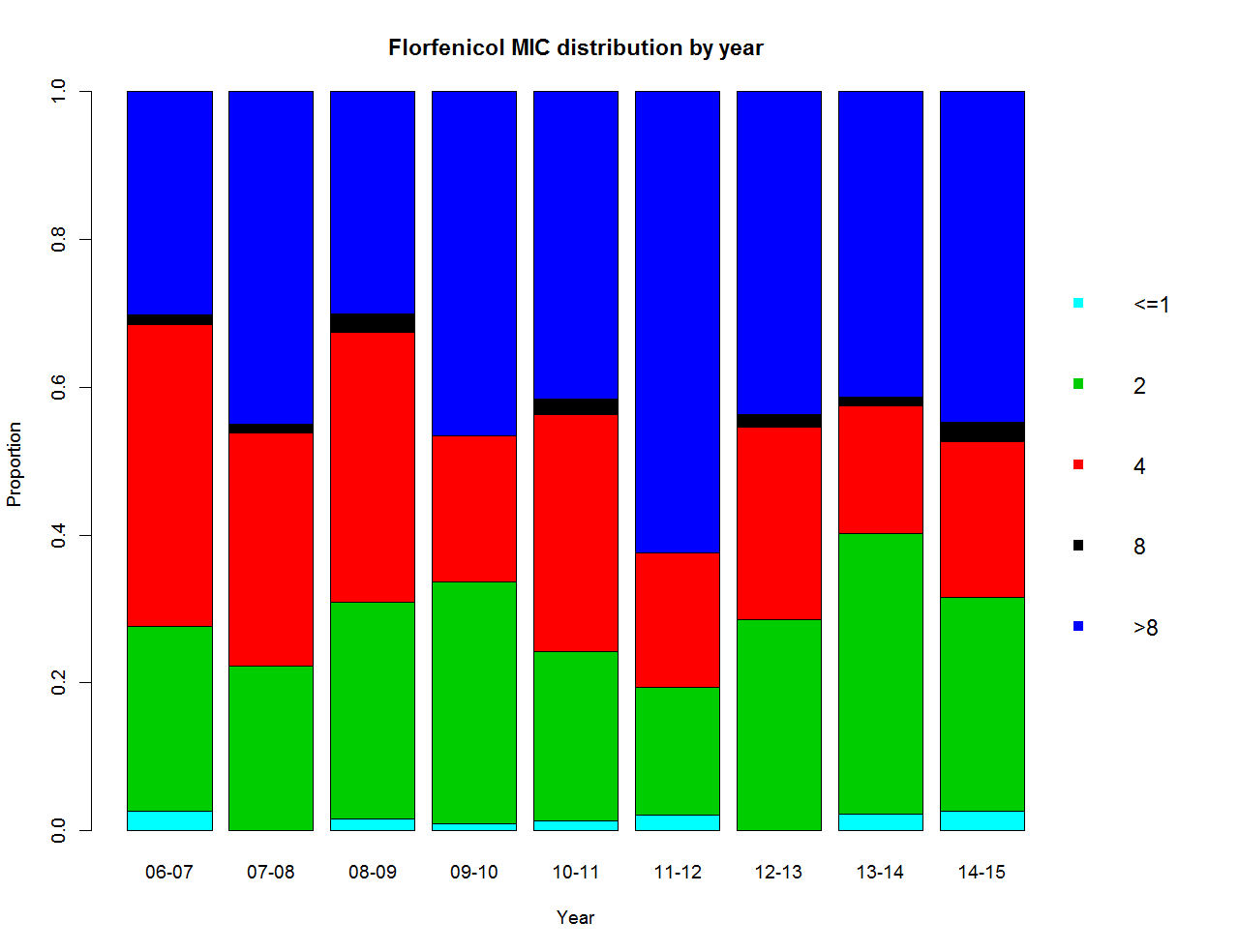

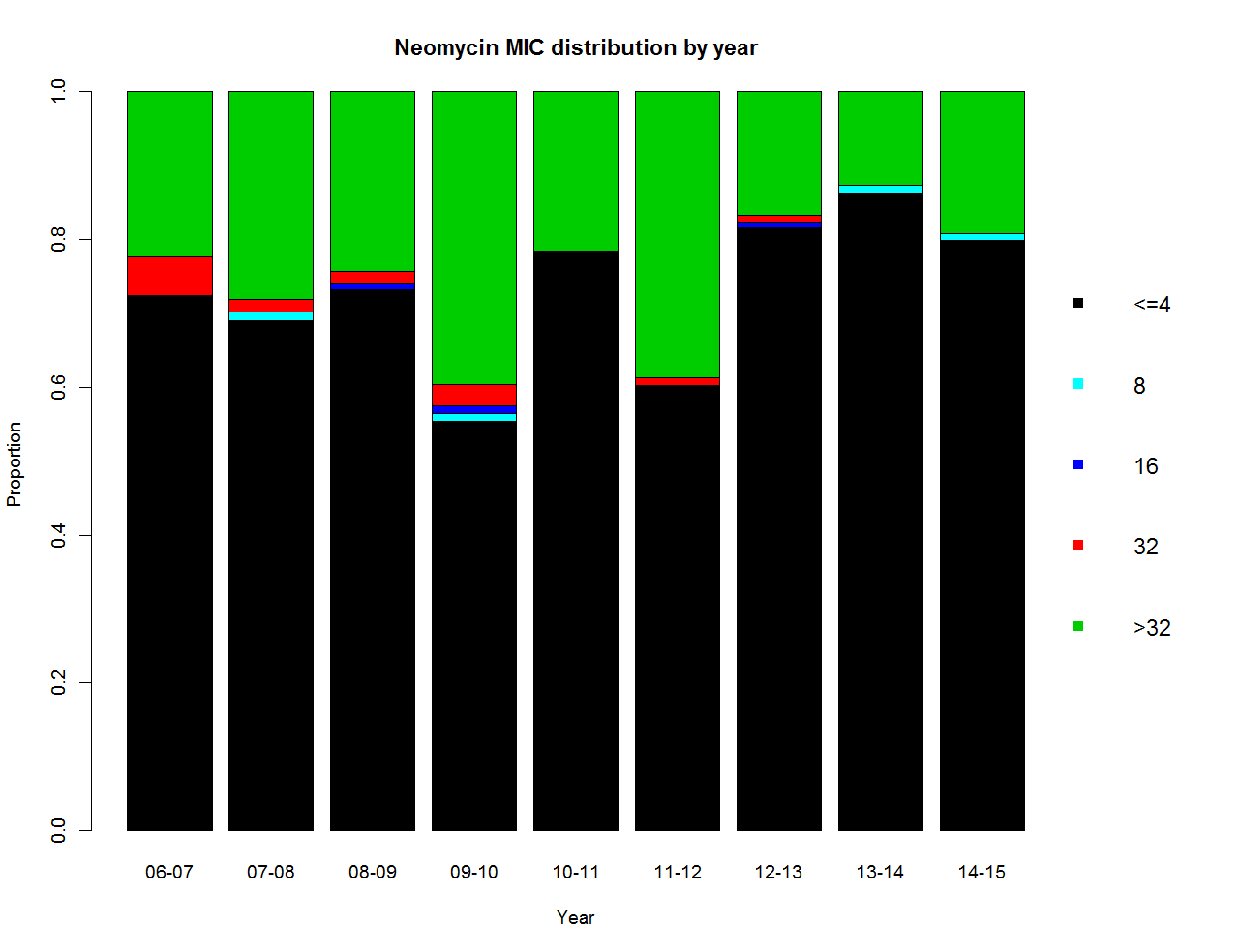

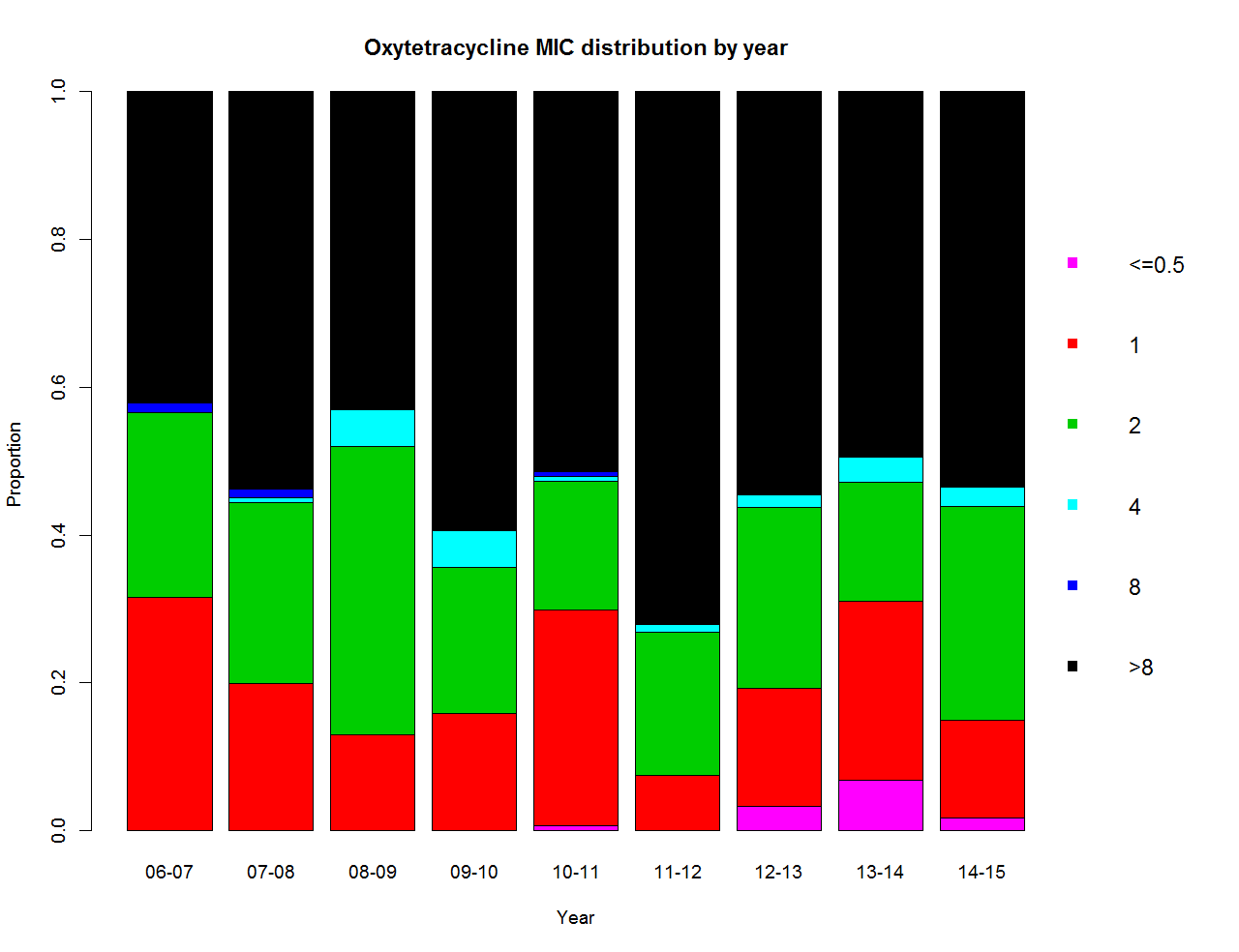

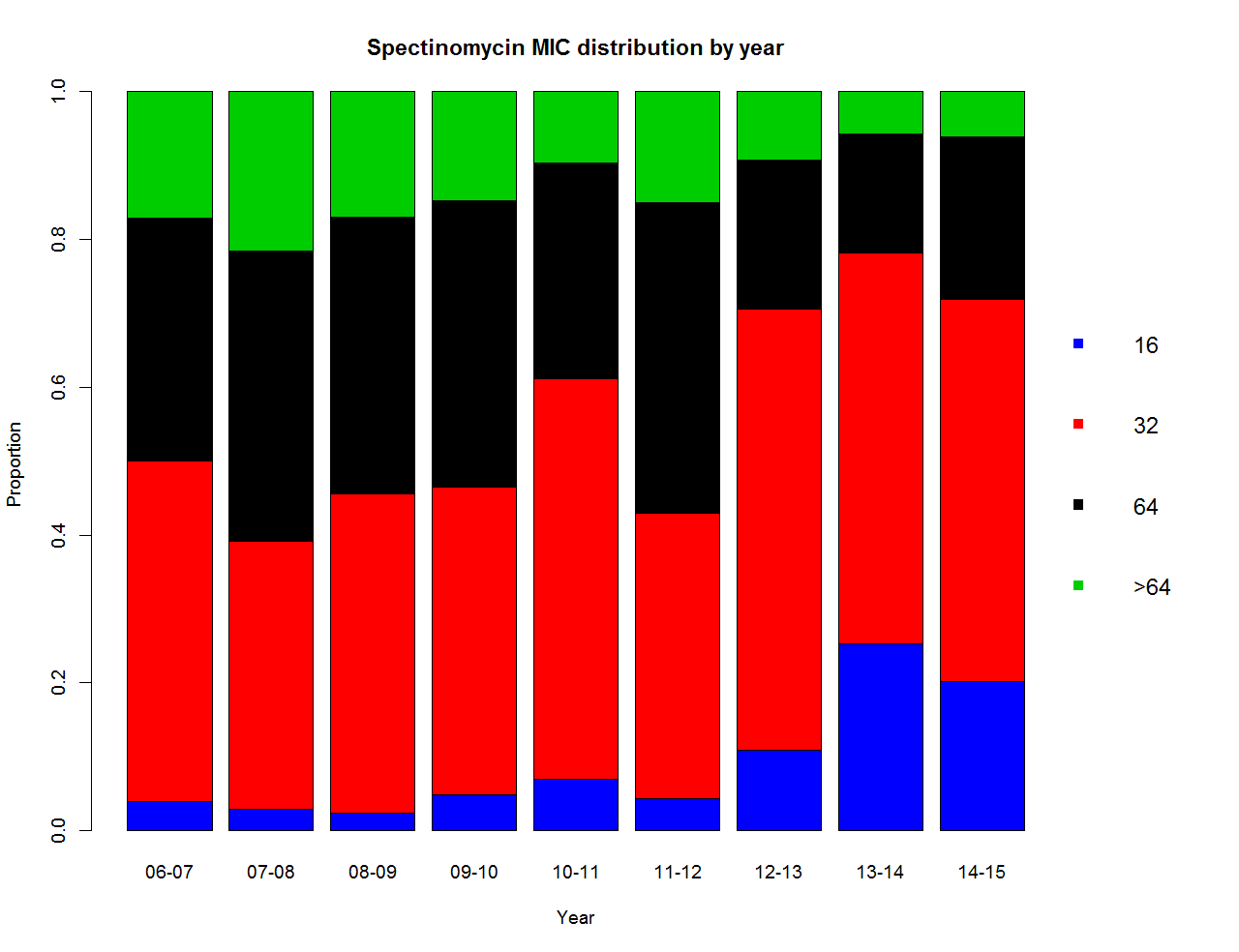

Supplement: S2 Fig — Distribution of the proportion of Salmonella isolates recovered from cattle showing each minimum inhibitory concentration (MIC) per year: 06–07, n = 76; 07–08, n = 171; 08–09, n = 123; 09–10, n = 101; 10–11, n = 144; 11–12, n = 93; 12–13, n = 119; 13–14, n = 87; 14–15, n = 114. (DOCX) [file pone.0168016.s002.docx]
